# A Survey of Primary Care Clinician Experiences With Electronic Health Record–Based Clinical Decision Support to Improve HIV Pre-Exposure Prophylaxis Prescribing

**DOI:** 10.2196/89638

**Published:** 2026-04-16

**Authors:** Afiba Manza-A Agovi, Caitlin T Thompson, Wentao Li, Jennifer Allard, Nicole Lee, Esther Fasanmi, Rohit P Ojha

**Affiliations:** 1 Center for Epidemiology & Healthcare Delivery Research, JPS Health Network, 1500 South Main Street, Fort Worth, TX, 76104, United States, 1 817 702 8201; 2 True Worth Clinic, JPS Health Network, Fort Worth, TX, United States; 3 Pharmacy Clinical Services Outpatient, JPS Health Network, Fort Worth, TX, United States

**Keywords:** clinical decision support systems, HIV pre-exposure prophylaxis, PrEP prescribing, sexual history documentation, primary care clinicians, community health centers, electronic health records, implementation science, physician engagement

## Abstract

A survey of primary care clinicians suggests that a clinical decision support tool to support sexual risk assessment and prescribing of HIV pre-exposure prophylaxis was appropriate and useful for identifying at-risk patients, but uptake was hindered by workflow and usability barriers, which underscores the importance of postimplementation clinician feedback to improve the use of clinical decision support tools.

## Introduction

Inadequate sexual history screening in US primary care limits identification of candidates for HIV pre-exposure prophylaxis (PrEP) and exacerbates inequities in PrEP uptake [1,2]. Many electronic health record (EHR) systems lack standardized prompts for documenting sexual risk, which contributes to missed opportunities for sexual health discussions and HIV prevention. This documentation gap is compounded by limited provider knowledge of PrEP and the absence of validated tools to guide prescribing decisions [3,4].

EHR-based clinical decision support (CDS) tools may address these gaps by facilitating sexual risk assessment and supporting PrEP prescribing [5]. Prior studies suggest that CDS tools are acceptable and feasible [6,7] and may improve PrEP prescribing [5]. Nevertheless, clinician experiences using CDS tools for sexual risk assessment and PrEP prescribing in primary care remain poorly understood. Clinician feedback about such CDS tools may inform efforts to optimize use of these tools. Therefore, we aimed to describe primary care clinicians’ experiences using CDS tools implemented in urban community health clinics to support sexual risk assessment and PrEP prescribing.

## Methods

### Study Design

We followed the Checklist for Reporting of Survey Studies (CROSS) guidelines to structure this report. We surveyed primary care clinicians from 12 community health clinics at John Peter Smith Health Network (JPS), a publicly funded urban health system that serves as the health care safety net for Tarrant County, Texas. JPS implemented an EHR-based CDS tool comprising a sexual history questionnaire, a PrEP SmartSet, and a noninterruptive advisory alert (Multimedia Appendix 1). When clinicians complete the sexual history questionnaire during an outpatient visit, a noninterruptive PrEP advisory alert is triggered if documented responses indicate behavioral or sexual risk factors consistent with US Centers for Disease Control and Prevention PrEP eligibility criteria.

### Survey Administration and Data Collection

We conducted a cross-sectional survey using a 12-item questionnaire to assess clinician awareness, use, perceptions, and perceived barriers and facilitators to the implementation of the CDS tool. Eligible participants included attending physicians, residents, nurse practitioners, physician assistants, and clinical pharmacists providing primary care at one of 12 JPS community health clinics. Survey items were adapted from prior CDS research and informed by the Consolidated Framework for Implementation Research (Multimedia Appendix 2). The anonymous web-based questionnaire (Research Electronic Data Capture) was distributed by email from July 8 to 29, 2024, and included 11 closed-ended items in Likert-scale format and one open-ended question to identify perceived implementation barriers and facilitators (Multimedia Appendix 3). The survey included core questions on tool awareness or use for all respondents and used branching (conditional) logic that prompted questions about tool perceptions only to respondents who reported awareness.

### Statistical Analysis

We summarized scores for closed-ended responses using medians and interquartile ranges (IQRs) and summarized frequencies of agreement with each item, where agreement reflects “agree” or “strongly agree” and disagreement reflects “disagree” or “strongly disagree.” Neutral responses were included in the denominator of these frequencies but not separately reported for easier contrast of extremes. We analyzed open-ended responses using conventional content analysis supported by a large language model (GPT-4 by ChatGPT) to identify recurring themes (Multimedia Appendix 4).

### Ethical Considerations

The North Texas Regional Institutional Review Board approved this study (number 2023‐151). This study followed the ethical standards of the responsible committee on human experimentation and the World Medical Association Declaration of Helsinki. All participants provided informed consent before participation. The survey was accessible only after consent was obtained. We collected responses anonymously and did not link any personally identifiable information to the survey data, ensuring privacy and confidentiality. Clinicians who completed the survey were entered into a draw for one of eight US $50 Walmart e-gift cards as compensation.

## Results

Our study population comprised 33 clinicians (12% of 284 eligible). Participant characteristics are summarized in Multimedia Appendix 5. Most respondents were physicians (MD/DO, n=20, 61%), followed by nurse practitioners (n=10, 30%). The majority were older than 30 years (n=29, 88%), identified as female (n=23, 70%), and were non-Hispanic White (n=19, 58%).

Fewer than half of respondents (n=15, 45%) were aware of the updated sexual history questionnaire. Table 1 summarizes awareness, use, and perceptions of the CDS tool among respondents who reported awareness of the tool. Most clinicians who reported awareness or use of the tool agreed that the questionnaire was important to implement (n=10, 67%), helpful for identifying PrEP candidates (n=9, 60%), and appropriate for primary care (n=9, 60%). In contrast, clinicians reported concerns about increased patient interaction time (n=9, 60%) and workflow fit (n=7, 47%). Most clinicians agreed that the PrEP advisory alert was appropriate for primary care (n=12, 78%) and supported guideline-concordant care (n=11, 73%), although fewer reported that the alert fit within clinical workflows (n=6, 40%).

Qualitative analysis of open-ended responses from 10 clinicians identified 5 themes: workflow disruption and time burden, alert fatigue, discomfort with the sexual history questionnaire, limited understanding of CDS functionality, and perceived usefulness for identifying at-risk patients. Table 2 summarizes illustrative quotes from these themes.

**Table 1.** Awareness, use, and perceptions of the CDS^a^ tool among clinicians who completed both awareness and use questions (N=15).^b^

	Median (IQR)	Agreement^c^, n (%)	Disagreement^c^, n (%)
Clinician attitudes and perceptions of the sexual history questionnaire			
I like this tool	3.0 (2.0‐4.0)	5 (33)	5 (33)
Suitable for my patients	4.0 (2.5‐4.0)	8 (53)	4 (27)
The tool is easy to use	3.0 (2.5‐4.0)	7 (47)	4 (27)
Trust evidence quality and validity	4.0 (3.0‐4.0)	9 (60)	2 (13)
Good option to identify sexual risk factors	3.0 (3.0‐4.0)	7 (47)	2 (13)
Facilitates sexual history-taking	3.0 (2.0‐4.0)	6 (40)	6 (40)
Meets needs to provide resources to patients	3.0 (2.5‐4.0)	5 (33)	4 (27)
Important to implement	4.0 (3.0‐4.0)	10 (67)	1 (6.7)
Appropriate for primary care	4.0 (3.0‐4.0)	9 (60)	3 (20)
Fits within workflow	3.0 (2.0‐4.0)	5 (33)	7 (47)
Does not increase patient time	2.0 (1.0‐3.0)	3 (20)	9 (60)
Easy to access and incorporate in workflow	3.0 (2.0‐4.0)	6 (40)	5 (33)
Valuable for primary care	4.0 (3.0‐4.0)	10 (67)	3 (20)
Helps identify PrEP^d^ candidates	4.0 (3.0‐4.0)	9 (60)	3 (20)
Clinician attitudes and perceptions of the PrEP advisory alert			
I like this alert	3.0 (3.0‐4.0)	7 (47)	1 (6.7)
Suitable for my patients	3.0 (3.0‐4.0)	7 (47)	2 (13)
Useful and actionable	3.0 (3.0‐4.0)	7 (47)	3 (20)
Trust evidence quality and validity	3.0 (3.0‐4.0)	7 (47)	3 (20)
Good for guideline-concordant care for PrEP	4.0 (3.5‐4.0)	11 (73)	0 (0)
Facilitates PrEP assessment	3.5 (3.0‐4.0)	7 (50)	2 (14)
Easy to access and incorporate in workflow	3.0 (2.5‐4.0)	6 (40)	4 (27)
Appropriate for primary care	4.0 (4.0‐4.0)	12 (79)	0 (0)
Fits within existing workflow	3.0 (2.5‐4.0)	6 (40)	4 (27)
Does not increase patient time	2.0 (2.0‐3.0)	2 (13)	8 (53)
Adds value to practice	4.0 (3.0‐4.0)	8 (53)	3 (20)
Valuable for primary care	4.0 (3.0‐4.0)	9 (64)	1 (6.7)
Helps identify PrEP candidates	4.0 (3.0‐4.0)	8 (53)	0 (0)

a CDS: clinical decision support.

b Clinician level of awareness and use frequency of the sexual history questionnaire: n=6, 40% aware, no use; n=9, 60% aware, used at least once.

c Agreement reflects “agree” or “strongly agree.” Disagreement reflects “disagree” or “strongly disagree.” Neutral responses were included in the denominator but not separately reported.

d PrEP: pre-exposure prophylaxis.

**Table 2.** Themes and illustrative quotes from clinician responses to open-ended questions regarding the use of the clinical decision support tool (sexual history questionnaire and PrEP^a^ advisory alert).

Theme	Illustrative quotes (participant ID)
Concern about time and workflow	The questionnaire is long; typical workflow is that MA [medical assistant] does questions, but then I do not see this information flagged for review if risks identified, so not sure if MA filling out correctly. (#3) It does take some time to complete the advanced sexual history and can be cumbersome if patient has other medical issues to discuss. (#10) Lack of time, patients and I both feel uncomfortable. I don’t even know how to find the questionnaire and was not aware I should be using it. (#11)
Time and reducing alert fatigue as a facilitator	If it doesn’t increase time during visits. There are already so many screenings we are doing. Many of the patients have multiple comorbid conditions that take time to address. The language and cultural barrier all add up to long visit times. (#14) Time. Along with all of the mandatory screening tools. I feel we ‘screen’ our patients to death. (#2) It might be easy to think that a BPA [Best Practice Alert] is helpful, and it may be, but we are subjected to easily 3 to 5 BPAs that each add minutes to our workflow for each patient we see. Please help us reduce time spent with a computer rather than add to it. (#15)
Patient and provider comfort and appropriateness as a barrier	There needs to be some sensitivity in how the questions are asked for refugee patients. I mostly work with refugees who are low risk. (#14) For the right patient history on initial screen this would be useful—not on every patient. (#6) This is not for every patient (#9) This can [be a] very intrusive questionnaire (#2)
Training	I have no experience prescribing PrEP and have not seen others do it. I feel uncomfortable with PreP. (#11) Training on tool...to understand how it is triggered. (#3)
Perceived utility of tool	The tool works well...if a patient is identified as being at risk for HIV based on the brief sexual screening, I use this tool. (#10) I think it is a good tool; however, our patients complete many required screening tools every time they come to the office... (#11) Useful for specific patients. (#6)

a PrEP: pre-exposure prophylaxis.

## Discussion

Our findings suggest suboptimal awareness of the CDS tool among primary care clinicians, and uptake was limited even among clinicians who reported awareness. Clinicians who used the tool generally endorsed the value of the sexual history questionnaire and PrEP advisory alert but reported concerns regarding usability and workflow integration, particularly increased time burden during patient visits. Open-ended responses revealed workflow disruption, alert fatigue, discomfort with the sexual history questionnaire, and limited understanding of tool functionality.

Our findings should be considered in the context of a key limitation. Despite a structured reminder strategy, financial incentives, and peer-led outreach, only 12% of eligible clinicians completed the survey, and thus our findings are likely sensitive to selection bias. Low response proportions are a persistent challenge when using provider surveys and may reflect competing clinical demands or survey fatigue [8]. We were unable to apply quantitative approaches to explore the effects of this selection bias [9] because we lacked data about clinicians who did not respond to the survey. Nevertheless, we likely overestimated CDS tool awareness among our primary care clinicians, and responses among tool users likely reflect the perspectives of a highly select group of early adopters, which could overestimate favorable impressions of the CDS tool compared with the overall eligible population.

Despite the key limitation, our findings align with prior studies of CDS implementation in primary care [10] that report low awareness and barriers related to workflow integration, time constraints, alert fatigue, and limited stakeholder engagement. Clinicians in our study endorsed the potential value of the CDS tool for identifying patients who may benefit from PrEP, but perceived workflow burdens may limit adoption. These findings reinforce the importance of aligning CDS tools with clinical workflows and eliciting postimplementation clinician feedback to improve the use of CDS tools.

## Supplementary material

10.2196/89638 Multimedia Appendix 1 Description of the clinical decision support intervention and screenshots of the sexual history questionnaire, pre-exposure prophylaxis advisory alert, and PrEP SmartSet in Epic.

10.2196/89638 Multimedia Appendix 2 Survey measures mapped to implementation constructs by Weiner et al [11] and the Consolidated Framework for Implementation Research (CFIR).

10.2196/89638 Multimedia Appendix 3 Description of the survey instrument assessing clinician perceptions and use of the pre-exposure prophylaxis clinical decision support tool.

10.2196/89638 Multimedia Appendix 4 ChatGPT input prompts used to support qualitative analysis.

10.2196/89638 Multimedia Appendix 5 Characteristics of primary care clinicians who responded to a survey on an electronic health record–based clinical decision support tool for pre-exposure prophylaxis, stratified by awareness of the intervention.

## Abbreviations

CDS: clinical decision support
CROSS: Checklist for Reporting of Survey Studies
EHR: electronic health record
IQR: interquartile range
JPS: John Peter Smith Health Network
PrEP: pre-exposure prophylaxis
