# Supplementary material for: A Survey of Primary Care Clinician Experiences With Electronic Health Record–Based Clinical Decision Support to Improve HIV Pre-Exposure Prophylaxis Prescribing
Source: JMIR Form Res. 2026 Apr 16;10:e89638. doi: 10.2196/89638 (PMC13085992; doi:10.2196/89638)
Supplement: Multimedia Appendix 1 [file formative-v10-e89638-s001.docx]

**Multimedia Appendix 1. Description of the CDS intervention and screenshots of the sexual history questionnaire, PrEP advisory alert, and PrEP SmartSet in Epic^a^.**

*Intervention description*

JPS implemented a locally developed EHR-based (Epic Systems, Verona, WI) CDS tool comprising a sexual history questionnaire, a PrEP SmartSet, and a non-interruptive advisory alert. The intervention was rolled out between September 2022 and January 2023. The sexual history questionnaire follows the U.S. Centers for Disease Control and Prevention Five Ps framework (i.e., partners, practices, protection, past sexually transmitted infections, and pregnancy prevention or intentions) to document sexual risk factors and identify potentially high-risk patients. When clinicians complete the sexual history questionnaire and patient responses indicate behavioral or sexual risk factors consistent with CDC PrEP eligibility criteria. A non-interruptive advisory alert prompts clinicians to consider PrEP prescribing and provides access to the PrEP SmartSet for documentation and laboratory ordering. The alert appears during the outpatient encounter and can be dismissed by documenting patient decline or initiating the SmartSet. Clinicians were not offered formal training for using these tools, but educational materials were posted on the health network intranet for clinicians to access.


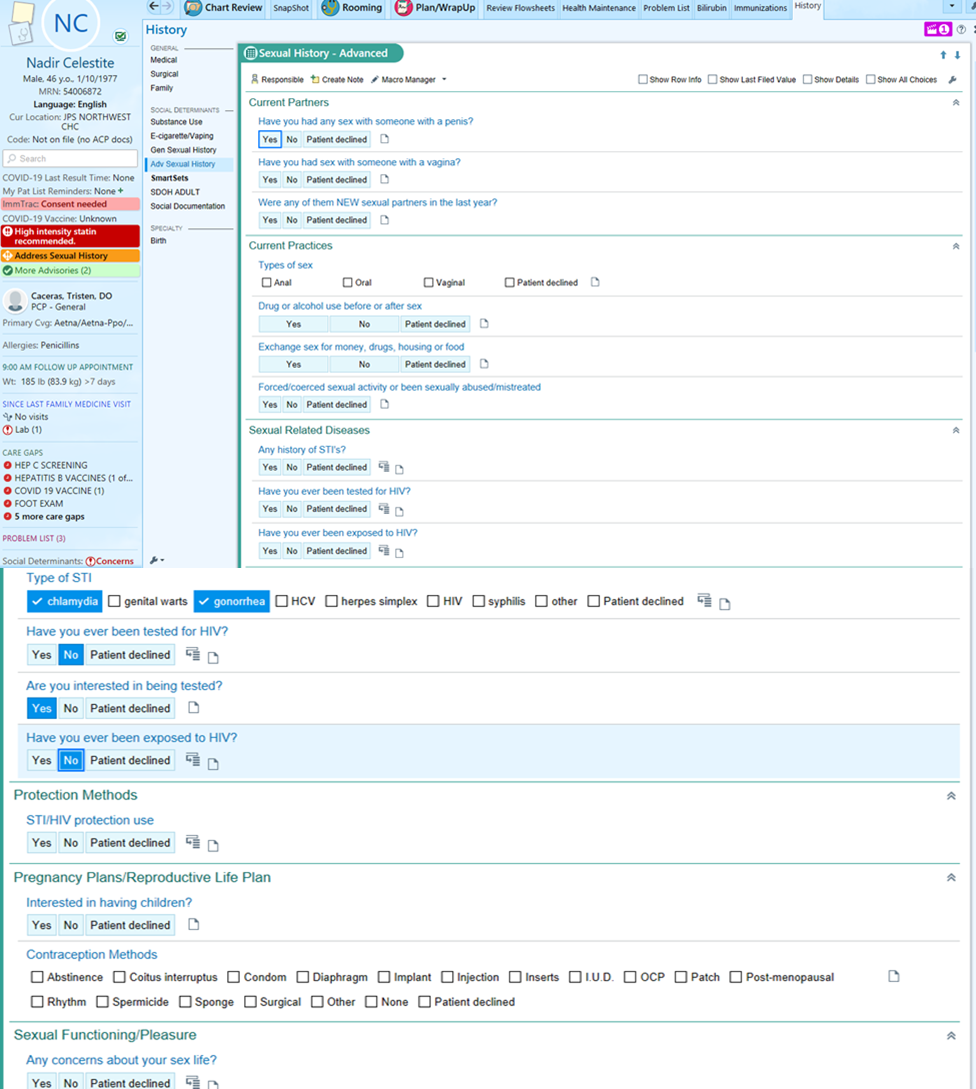


^a^Screenshot for demonstration purposes only. Test patient and physician. No protected health information is included.


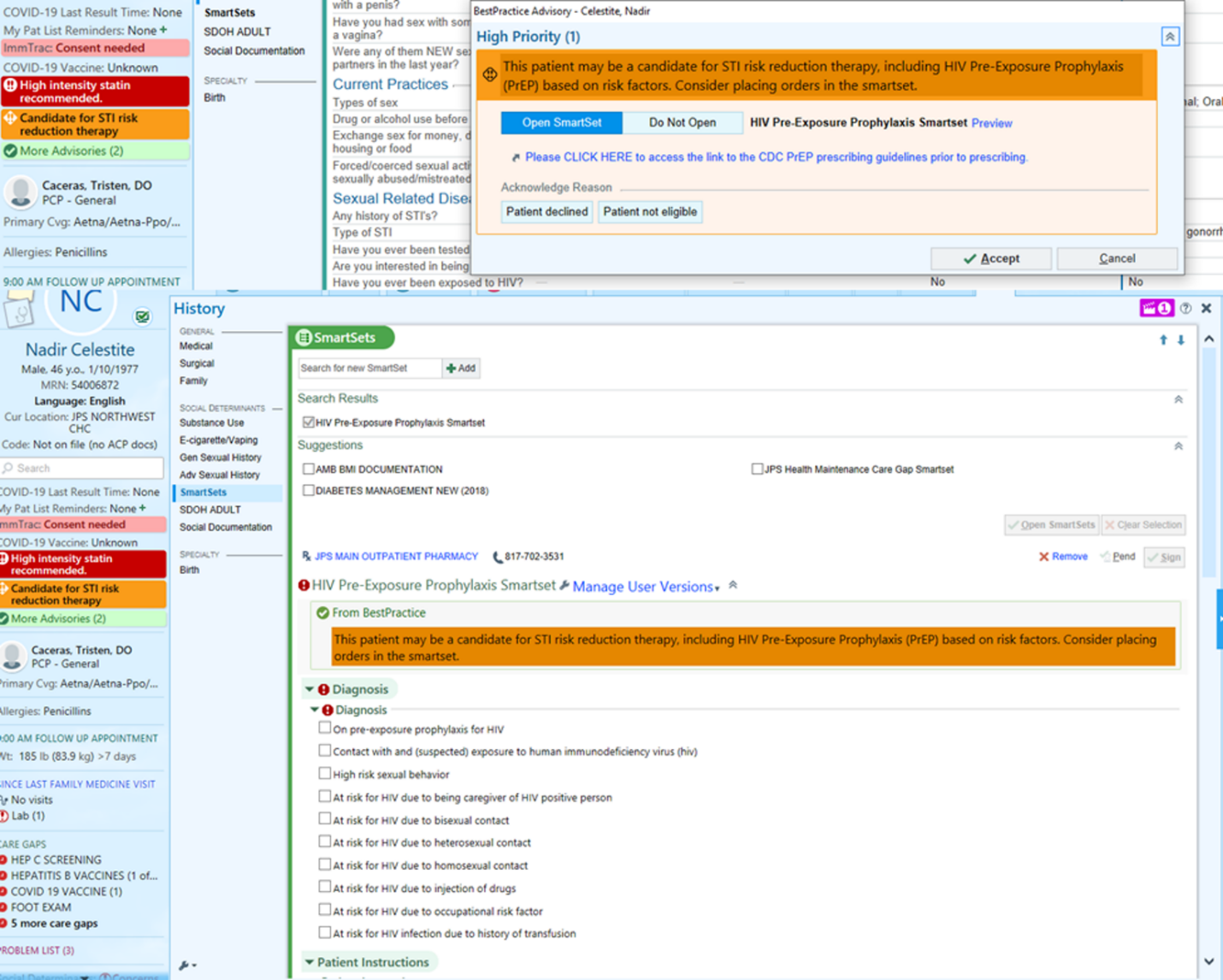


^a^Screenshot for demonstration purposes only. Test patient and physician. No protected health information is included.
