# Supplementary material for: A Survey of Primary Care Clinician Experiences With Electronic Health Record–Based Clinical Decision Support to Improve HIV Pre-Exposure Prophylaxis Prescribing
Source: JMIR Form Res. 2026 Apr 16;10:e89638. doi: 10.2196/89638 (PMC13085992; doi:10.2196/89638)
Supplement: Multimedia Appendix 2 [file formative-v10-e89638-s002.docx]

**Multimedia Appendix 2. Survey measures^1^ mapped to implementation constructs by Weiner et al. and the Consolidated Framework for Implementation Research (CFIR).**

| Construct/measures assessed | | Survey Questions^1^ |
| --- | --- | --- |
| Acceptability of intervention measure | | 1. This tool meets my approval |
| Intervention appropriateness measure | | 1. This tool seems fitting and suitable for my patients |
| Feasibility of intervention measure | | 1. This tool is easy to use |
| CFIR – Intervention characteristics | Evidence & strength | 1. I trust the quality and validity of evidence supporting this intervention |
|  | Relative advantage | 1. Implementing this tool is a good option to identify patients with sexual risk factors at JPS |
|  | Relative advantage | 1. This tool facilitates how I obtain sexual histories from my patients |
| CFIR – Outer setting | Patient Needs and Resources | 1. This tool meets my needs to provide needed resources to my patients |
| CFIR – Inner setting | Relative priority | 1. I recognize the importance of implementing this tool in practice |
|  | Compatibility | 1. This tool is appropriate for clinicians in primary care |
|  | Compatibility | 1. This tool fits within my existing workflow |
|  | Compatibility | 1. This tool does not increase the time needed with a patient |
|  | Access to knowledge and information | 1. This tool is easy to access and incorporate into my workflow |
| Characteristics of individuals | Knowledge and Beliefs about intervention | 1. This tool is valuable for clinicians in primary care |
|  | Self -efficacy | 1. This tool helps me to identify patients who may benefit from HIV PrEP |

^1^ Adapted from Bangash H, Pencille L, Gundelach JH, Makkawy A, Sutton J, Makkawy L, Dikilitas O, Kopecky S, Freimuth R, Caraballo PJ, Kullo IJ. An Implementation Science Framework to Develop a Clinical Decision Support Tool for Familial Hypercholesterolemia. J Pers Med. 2020 Jul 23;10(3):67. doi: 10.3390/jpm10030067. PMID: 32717811; PMCID: PMC7565418.
