# Supplementary material for: A Survey of Primary Care Clinician Experiences With Electronic Health Record–Based Clinical Decision Support to Improve HIV Pre-Exposure Prophylaxis Prescribing
Source: JMIR Form Res. 2026 Apr 16;10:e89638. doi: 10.2196/89638 (PMC13085992; doi:10.2196/89638)
Supplement: Multimedia Appendix 3 [file formative-v10-e89638-s003.docx]

**Multimedia Appendix 3. Description of the survey instrument assessing clinician perceptions and use of the PrEP CDS tool**.

*Survey instrument and administration*

We developed a 12-item, English language questionnaire (*Multimedia Appendix 2*) comprising 11 closed-ended items to assess clinicians’ awareness, perceptions, and use of the CDS intervention, and one open-ended item to elicit perceived barriers and facilitators. We adapted survey questions from a prior study that used the Acceptability of Intervention Measure, Intervention Appropriateness Measure, and Feasibility of Intervention Measure, along with constructs from the Consolidated Framework for Implementation Research, to assess clinicians’ perceptions of a CDS tool and contextual factors influencing implementation. *Multimedia Appendix 3* maps survey items to implementation constructs. We incorporated branching logic (conditional) to reflect varying levels of clinician engagement with the CDS tool. Clinicians rated statements about the updated sexual history questionnaire and the PrEP advisory alert using a 5-point Likert scale (1 = Strongly disagree, 5 = Strongly agree). The open-ended item invited clinicians to describe challenges using the tools in Epic and to identify the single most important factor that would facilitate tool use. Demographic items included age, sex, race/ethnicity, practice specialty, primary degree, and years since residency completion. The estimated completion time for this survey was 10 minutes.

We identified eligible participants from a clinician email list provided by the JPS Medical Staff Office and invited clinicians to complete the survey by email. One week before survey launch, a peer physician-leader sent a preparatory email to eligible participants. We administered the anonymous, web-based survey using REDCap from July 8 to 29, 2024. The initial invitation email included a link to a consent statement and survey. Eligible participants received three reminder emails on days 5 and 14, and 21. Each reminder described survey procedures and included a survey link. Participants who completed the survey were entered into a drawing for one of eight $50 Walmart electronic gift cards.

**Instructions:** *The purpose of this survey is to gather responses from JPS clinicians on their attitudes and use of a sexual history-taking tool and CDS alert that was implemented in the EHR. This questionnaire is to be completed only by JPS PCMH clinicians with prescriptive authority.*

**Section 1: Use{Gagliardi, 2019 #19}{Gagliardi, 2019 #19}{Gagliardi, 2019 #19} of Sexual History- Advanced Questionnaire**

1. What is your level of awareness and use of the **Sexual History-Advanced Questionnaire** in Epic?

*Choose the response that best matches your scenario*

❑ I regularly use the **Sexual History-Advanced Questionnaire**

❑ I have used the **Sexual History-Advanced Questionnaire** a few times

❑ I have used the **Sexual History-Advanced Questionnaire** on one occasion then never again

❑ I am aware of the **Sexual History-Advanced Questionnaire** but have not used it

❑ I am aware of the **Sexual History-Advanced Questionnaire** but do not plan to use it

❑ I was not aware of the **Sexual History-Advanced Questionnaire** prior to this survey

1. How often do you use the **Sexual History-Advanced Questionnaire** to help guide your decisions about discussing PrEP for HIV prevention?

❑ Always (>90%)

❑ Very Often (75-90%)

❑ Often (50-75%)

❑ Sometimes (25-50%)

❑ Rarely (<25%)

**Section 2: Attitudes Toward and Perceptions of the Sexual History Advanced Questionnaire**

1. Please indicate your level of agreement with the following statements concerning your experience with the **Sexual History-Advanced Questionnaire in Epic.** Select **one** answer in each row that is closest to your viewpoint.

|  | **Strongly Disagree** | **Disagree** | **No opinion** | **Agree** | **Strongly Agree** |
| --- | --- | --- | --- | --- | --- |
| 1. I like this tool |  |  |  |  |  |
| 1. This tool seems fitting and suitable for my patients |  |  |  |  |  |
| 1. This tool is/seems easy to use |  |  |  |  |  |
| 1. I trust the quality and validity of evidence supporting this intervention |  |  |  |  |  |
| 1. Implementing this tool is a good option to identify patients with sexual risk factors at JPS |  |  |  |  |  |
| 1. This tool facilitates how I obtain sexual histories from my patients |  |  |  |  |  |
| 1. This tool meets my needs to provide needed resources to my patients |  |  |  |  |  |
| 1. I recognize the importance of implementing this tool in practice |  |  |  |  |  |
| 1. This tool is appropriate for clinicians in primary care |  |  |  |  |  |
| 1. This tool fits within my existing workflow |  |  |  |  |  |
| 1. This tool does not increase the time needed with a patient |  |  |  |  |  |
| 1. This tool is /appears easy to access and incorporate into my workflow |  |  |  |  |  |
| 1. This tool is valuable for clinicians in primary care |  |  |  |  |  |
| 1. This tool helps me to identify patients who may benefit from HIV PrEP |  |  |  |  |  |

1. a. Please tell us any additional thoughts or challenges about your experience with using the **Sexual History-Advanced Questionnaire in Epic?**

|  |
| --- |

1. b. What is the single most important factor that enables or will enable your use of **Sexual History-Advanced Questionnaire in Epic**?

|  |
| --- |

**Section 3: Attitudes and Perceptions of the Best Practice Advisory Alert for HIV PrEP**

1. To what extent do you agree with the following statements concerning your experience with the **Best Practice Advisory Alert for HIV PrEP in Epic?**  Select **one** answer in each row that is closest to your viewpoint.

|  | **Strongly Disagree** | **Disagree** | **No opinion** | **Agree** | **Strongly Agree** |
| --- | --- | --- | --- | --- | --- |
| 1. I like this advisory alert for HIV PrEP |  |  |  |  |  |
| 1. This advisory alert for HIV PrEP seems fitting and suitable for my patients |  |  |  |  |  |
| 1. This advisory alert for HIV PrEP is useful and actionable |  |  |  |  |  |
| 1. I trust the quality and validity of evidence supporting the advisory alert for HIV PrEP |  |  |  |  |  |
| 1. Implementing this PrEP advisory alert is a good option to provide guideline concordant care for PrEP at JPS |  |  |  |  |  |
| 1. This advisory alert facilitates how I evaluate patients for PrEP candidacy |  |  |  |  |  |
| 1. This advisory alert is easy to access and incorporate into my workflow |  |  |  |  |  |
| 1. This alert is appropriate for clinicians in primary care |  |  |  |  |  |
| 1. This alert fits within my existing workflow |  |  |  |  |  |
| 1. This alert does not increase the time needed with a patient |  |  |  |  |  |
| 1. Responding to this advisory alert for PrEP adds an extra burden to practice, for me or my patients (i.e. time, costs, etc.) |  |  |  |  |  |
| 1. This advisory alert is valuable for clinicians in primary care |  |  |  |  |  |
| 1. This alert helps me to identify patients who may benefit from HIV PrEP use |  |  |  |  |  |

1. a. Please tell us any additional thoughts or challenges concerning your experience with the **Best Practice** **Advisory Alert for HIV PrEP** in Epic?

|  |
| --- |

b. What is the single most important factor that will enhance your experience interacting with the **Best Practice** **Advisory Alert for HIV PrEP** in Epic?

|  |
| --- |

**Section 4: Demographic Information**

| 1. **Sex**   ❑ Female ❑ Male  ❑ Prefer not to respond | 1. **Age**   ❑ ≤30 ❑ 31 – 40 ❑ 41– 50 ❑ 51 – 60 ❑ >60 |
| --- | --- |
| 1. **Race/Ethnicity**   ❑ White, Non-Hispanic  ❑ Black, Non-Hispanic  ❑ Hispanic  ❑ Asian  ❑ Other, please specify _______  ❑ Prefer not to respond | 1. **Primary area of practice /Specialty:**   ❑ Infectious disease  ❑ Family medicine  ❑ Internal medicine  ❑ Obstetrician/Gynecologist  ❑ Other, please specify ________________ |
| 1. **Years of clinical practice after residency:**   ❑ < 5 years  ❑ 5-9 years  ❑ 10-14 years  ❑ 15-19 years  ❑ ≥ 20 years | 1. **Primary degree/specialty**   ❑ Primary care Physician (MD/DO)  ❑ Nurse Practitioner (NP)  ❑ Clinical Pharmacist (PharmD)  ❑ Physician Assistant (PA)  ❑ Infectious disease specialist (MD/DO)  ❑ Other, please specify __________ |
