# Supplementary material for: A Survey of Primary Care Clinician Experiences With Electronic Health Record–Based Clinical Decision Support to Improve HIV Pre-Exposure Prophylaxis Prescribing
Source: JMIR Form Res. 2026 Apr 16;10:e89638. doi: 10.2196/89638 (PMC13085992; doi:10.2196/89638)
Supplement: Multimedia Appendix 4 [file formative-v10-e89638-s004.docx]

**Multimedia Appendix 4. ChatGPT input prompts used to support qualitative analysis.**

The following prompt was used to guide ChatGPT in conducting a conventional content analysis of clinician survey responses:

- You are a researcher analyzing qualitative data from survey responses. Use the attached qualitative data for analysis. Your task is to conduct a conventional content analysis by analyzing survey responses and extracting themes and differences found within the responses. I want you to follow these steps:
- Read the entire text multiple times to gain a general understanding of its content.
- Group text by Q4a, Q6a, Q4b, and Q6b, which are in columns B, C, D, and E.
- Divide the text into meaningful units, extracting summaries of meaningful units, and coding.
- Classify initial codes into categories and subcategories based on comparing similarities and differences.
- Extract themes as representations of concepts and hidden content in the data. I want you to specifically mention the respondent numbers, e.g., Respondent 4, 23, etc. When making comparisons, explicitly mention how their responses are similar to or different from one another. Be specific in the comparison.
- What are the key emerging themes? What are the results for the question "What is the single most important factor that enables or will enable your use of the Sexual History-Advanced-Questionnaire in Epic?"
- What are the results for this question: Please tell us any additional thoughts or challenges concerning your experience with the Best Practice Advisory Alert for HIV PrEP in Epic.
- What are the results for this question: What is the single most important factor that will enhance your experience interacting with the Best Practice Advisory Alert for HIV PrEP in Epic?
- Provide an overall thematic summary across all four questions.
