# Supplementary material for: A Survey of Primary Care Clinician Experiences With Electronic Health Record–Based Clinical Decision Support to Improve HIV Pre-Exposure Prophylaxis Prescribing
Source: JMIR Form Res. 2026 Apr 16;10:e89638. doi: 10.2196/89638 (PMC13085992; doi:10.2196/89638)
Supplement: Multimedia Appendix 5 [file formative-v10-e89638-s005.docx]

**Multimedia Appendix 5. Characteristics of primary care clinicians who responded to a survey on an EHR-based CDS tool for PrEP, stratified by awareness of the intervention.**

|  | Total | No Awareness^1^ | Aware^1^ |
| --- | --- | --- | --- |
| **Characteristic** | ***n* = 33** | ***n* = 18** | ***n* = 15** |
| **Sex; *n* (%)** |  |  |  |
| Female | 23 (70%) | 13 (72%) | 10 (67%) |
| Male | 7 (21%) | 5 (28%) | 2 (13%) |
| **Age, years; *n* (%)** |  |  |  |
| ≤ 30 | 4 (12%) | 2 (11%) | 2 (13%) |
| 31 – 40 | 11 (33%) | 6 (33%) | 5 (33%) |
| 41 – 50 | 10 (30%) | 6 (33%) | 4 (27%) |
| 51 – 60 | 8 (24%) | 4 (22%) | 4 (27%) |
| **Race/Ethnicity; *n* (%)** |  |  |  |
| Non-Hispanic White | 19 (58%) | 10 (56%) | 9 (60%) |
| Hispanic | 5 (15%) | 3 (17%) | 2 (13%) |
| Asian | 4 (12%) | 3 (17%) | 1 (6.7%) |
| **Provider Primary Degree; *n* (%)** |  |  |  |
| Clinical Pharmacist (PharmD) | 1 (3.0%) | 1 (5.6%) | 0 (0%) |
| Doctor of Medicine (MD/DO) | 20 (61%) | 10 (56%) | 10 (67%) |
| Nurse Practitioner (NP) | 10 (30%) | 6 (33%) | 4 (27%) |
| Physician Assistant (PA) | 2 (6.1%) | 1 (5.6%) | 1 (6.7%) |
| **Primary Area of Practice; *n* (%)** |  |  |  |
| Family medicine | 21 (64%) | 12 (67%) | 9 (60%) |
| Infectious disease | 1 (3.0%) | 1 (5.6%) | 0 (0%) |
| Internal medicine | 3 (9.1%) | 1 (5.6%) | 2 (13%) |
| Obstetrician/Gynecologist | 3 (9.1%) | 0 (0%) | 3 (20%) |
| Pediatrics | 2 (6.1%) | 2 (11%) | 0 (0%) |
| Primary care | 3 (9.1%) | 2 (11%) | 1 (6.7%) |
| **Years in clinical practice; *n* (%)** |  |  |  |
| < 5 | 13 (39%) | 8 (44%) | 5 (33%) |
| 5 – 9 | 2 (6.1%) | 1 (5.6%) | 1 (6.7%) |
| 10 – 14 | 9 (27%) | 5 (28%) | 4 (27%) |
| 15 – 19 | 5 (15%) | 2 (11%) | 3 (20%) |
| ≥ 20 | 4 (12%) | 2 (11%) | 2 (13%) |

PrEP, pre-exposure prophylaxis; CDS, clinical decision support; EHR, electronic health record.

^1^ Clinician awareness of the Advanced Sexual History–taking tool in Epic.
